# Cytoprotective Effect of Astaxanthin in a Model of Normal Intraocular Pressure Glaucoma

**DOI:** 10.1155/2020/9539681

**Published:** 2020-09-19

**Authors:** Kasumi Kikuchi, Zhenyu Dong, Yasuhiro Shinmei, Miyuki Murata, Atsuhiro Kanda, Kosuke Noda, Takayuki Harada, Susumu Ishida

**Affiliations:** ^1^Laboratory of Ocular Cell Biology & Visual Science, Department of Ophthalmology, Faculty of Medicine and Graduate School of Medicine, Hokkaido University, Sapporo, Japan; ^2^Visual Research Project, Tokyo Metropolitan Institute of Medical Science, Tokyo, Japan

## Abstract

Glaucoma is characterized by axonal degeneration of retinal ganglion cells (RGCs) and apoptotic death of their cell bodies. Lowering intraocular pressure is currently the only way to treat glaucoma, but it is often insufficient to inhibit the progression of the disease. Glaucoma is a multifactorial disease, and the involvement of oxidative stress has recently received much attention. In the present study, we investigated the cytoprotective effect of astaxanthin (AST) on RGC degeneration using a normal-tension glaucoma (NTG) mouse model, which lacks the *glutamate/aspartate transporter* (Glast) and demonstrates spontaneous RGC and optic nerve degeneration without elevated intraocular pressure. Three-week-old Glast^±^ mice were given intraperitoneal injections of AST at 10, 30, or 60 mg/kg/day or vehicle alone, and littermate control mice were given vehicle alone for 14 days, respectively. Five weeks after birth, the number of RGCs was counted in paraffin sections of retinal tissues stained with hematoxylin and eosin. We also used a retrograde labeling technique to quantify the number of RGCs. Additionally, the phosphorylated (p) I*κ*B/total I*κ*B ratio and the 4-hydroxynonenal (HNE) were measured in retinal tissues. The number of RGCs in Glast^±^ mice was significantly decreased compared with that in control mice. RGC loss was suppressed by the administration of AST at 60 mg/kg/day, compared with vehicle alone. Following AST administration, the concentration of 4-HNE in the retina was also suppressed, but the pI*κ*B/I*κ*B ratio did not change. Our study revealed that the antioxidative stress effects of AST inhibit RGC degeneration in the retina and may be useful in the treatment of NTG.

## 1. Introduction

Glaucoma is one of the leading causes of blindness worldwide [1, 2], characterized by axonal degeneration of retinal ganglion cells (RGCs) and apoptotic death of their cell bodies. Typically, glaucoma is associated with chronic elevation of intraocular pressure (IOP), and lowering IOP is associated with an attenuation of progressive optic nerve damage [3–5]. The main goal in glaucoma therapy is controlling IOP to a target level by medical therapy and parasurgical and surgical approaches in order to prevent or stop the loss of visual field [6]. Nevertheless, a growing body of evidence has demonstrated that IOP reduction alone is not sufficient to inhibit the progression of the disease [7], which suggests the contribution of other factors in the pathogenesis of glaucoma. In Japan, where normal-tension glaucoma (NTG) comprises the majority of glaucoma [8], it is of special importance to elucidate IOP-independent factors and explore alternative therapeutic strategies such as cytoprotection of RGCs.

Dysfunction of the glutamate/aspartate transporter (GLAST), one of the glutamate transporters, in gene-deficient mice has been shown to induce RGC death due to increased oxidative stress despite normal IOP [9–11]. The neurotoxicity of glutamate and oxidative stress are well known to be involved in retinal damage in a variety of ocular diseases, including glaucoma. Indeed, decreased glutamine transporter and glutathione levels have been detected in the retinas and plasma of glaucoma patients [12, 13]. Therefore, GLAST mutant mice are now widely used as an animal model for NTG [9–11].

Astaxanthin (AST) is a naturally occurring carotenoid whose structural and functional characteristics make it a promising bioactive compound for the prevention of several human diseases, as well as the maintenance of good health [14]. It belongs to the family of xanthophylls and is especially common in marine environments where it can be observed as a red-colored pigment that contributes to the pinkish-red color of salmonids, shrimps, lobsters, and crayfish's flesh [15]. Once biosynthesized by phytoplankton and microalgae, such as *Haematococcus pluvialis*, *Chlorella zofingiensis*, and *Xanthophyllomyces dendrorhous*, it accumulates in various aquatic species and represents the main dietary source of valuable nutrients. Recently, increasing evidence has suggested the efficacy of AST in the prevention and treatment of several ocular diseases, ranging from the anterior to the posterior poles of the eyes, via suppression of oxidative stress and inflammation [16].

In the present study, we investigated the cytoprotective effect of AST on RGC loss using Glast heterozygous knockout (Glast^±^) mice.

## 2. Materials and Methods

### 2.1. Animals and Reagents

Three-week-old C57BL/6J mice (WT; CLEA Japan, Tokyo, Japan) and Glast^±^ mice [9–11] were used in this study. There was no gender bias in each experimental group. The rodents were housed in the animal facility at Hokkaido University. Standard chow and water were provided *ad libitum*. All animal experiments were conducted following the guidelines of the Association for Research in Vision and Ophthalmology (ARVO) Statement for the Use of Animals in Ophthalmic and Vision Research. The experiment was approved by the Ethics Review Committee for Animal Experimentation of Hokkaido University (#18-0087). AST was purchased from FUJIFILM Wako Pure Chemical Corporation (Osaka, Japan). Anesthesia was induced by intraperitoneal injection of pentobarbital (0.05 mg/g body weight).

### 2.2. Histological and Morphometric Analyses

At the end of the 5^th^ week (i.e., following the 14-day administration of AST or vehicle), mice were euthanized with an overdose of anesthesia. The eyes were enucleated from the mice following fixation by intracardiac perfusion of 4% paraformaldehyde in PBS. Paraffin sections (5-*μ*m thick) of retinal specimens were cut through the optic nerve and stained with hematoxylin and eosin (H & E). The number of neurons in the ganglion cell layer (GCL) was counted from one ora serrata through the optic nerve to the other ora serrata, and the results of three serial sections were averaged for each sample in each group. Six to nine eyes were used from each group.

RGCs were retrogradely labeled from the superior colliculus with Fluoro-Gold (Biotium, Hayward, CA), and RGC density was determined as previously described [9–11]. Briefly, four standard areas (0.04 mm^2^) of each whole-mounted retina at the point of 0.1 mm from the optic disc were randomly chosen, and labeled cells were counted.

### 2.3. Measurement of the pI*κ*B/I*κ*B Ratio in the Retina

After 14 days of AST administration, the phosphorylated (p) I*κ*B/total I*κ*B ratio in the retina was measured using the PathScan® Phospho-I*κ*B*α* (Ser32) Sandwich ELISA Kit (Cell Signaling Technology, Danvers, MA) and PathScan® Total I*κ*B*α* Sandwich ELISA Kit (Cell Signaling Technology), respectively. Seven to eight eyes from four animals were used for each group. Briefly, retinas were collected from the mice following 14 days of AST administration, and proteins were extracted from the retina using a cell lysis buffer (Cell signaling Technology). The concentrations of pI*κ*B and I*κ*B were measured in accordance with the protocols of the ELISA kits mentioned above, respectively, and the pI*κ*B/I*κ*B ratio was calculated.

### 2.4. Determination of the 4-HNE Level in the Retina

Retinas were isolated under a microscope from WT mice and Glast^±^ mice treated with vehicle or AST at the end of 5 weeks of age. Protein concentration and 4-HNE levels in the retina were determined by BCA protein assay (Thermo Fisher Scientific, Waltham, MA) and the OxiSelect 4-HNE adduct competitive ELISA kit (Cell Biolabs, City, Country), respectively. Eight eyes from four animals were used for each group.

### 2.5. Statistical Analysis

All results are expressed as the mean ± standard error and n-numbers are as indicated. Student's *t*-tests were used for statistical comparison between groups. Differences between the means were considered significant when the probability values were <0.05.

## 3. Results

### 3.1. Inhibition of RGC Loss by AST Administration

**Figure 1:**
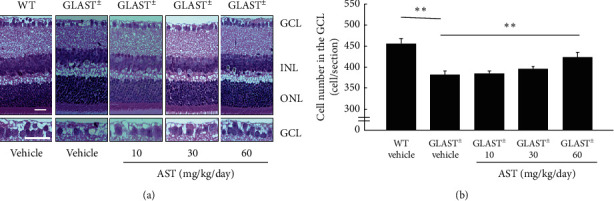
Dose-dependent inhibition of RGC loss by AST administration. (a) H & E staining of retinal sections. GCL: ganglion cell layer; INL: inner nuclear layer; ONL: outer nuclear layer. Scale bars: 25 *μ*m. (b) Quantification of cell number per section in the GCL. ^*∗∗*^*P* < 0.01.

RGC degeneration in the retina of Glast^±^ mice starts at 3 weeks of age [9]. To determine whether AST inhibits RGC loss, 3-week-old mice received intraperitoneal administration of AST at 10, 30, or 60 mg/kg/day or vehicle alone for 14 days (Figure 1(a)). Following the 14-day administration (i.e., at the end of the 5^th^ week), the number of RGCs in Glast^±^ mice was significantly decreased (382 ± 9/section; *n* = 8) compared with that in WT mice (456 ± 1/section; *n* = 9. *P* < 0.01; Figure 1(b)). RGC loss was significantly suppressed by AST administration at 60 mg/kg/day (425 ± 11/section; *n* = 8. *P* < 0.01; Figure 1(b)), while AST at 30 mg/kg/day (395 ± 6, *n* = 6) and 10 mg/kg/day (385 ± 6/section; *n* = 8) administration did not suppress the loss of RGCs. These results indicate that AST administration protects against RGC degeneration.

### 3.2. AST-Mediated Protection of RGCs Detected by Retrograde Labeling

**Figure 2:**
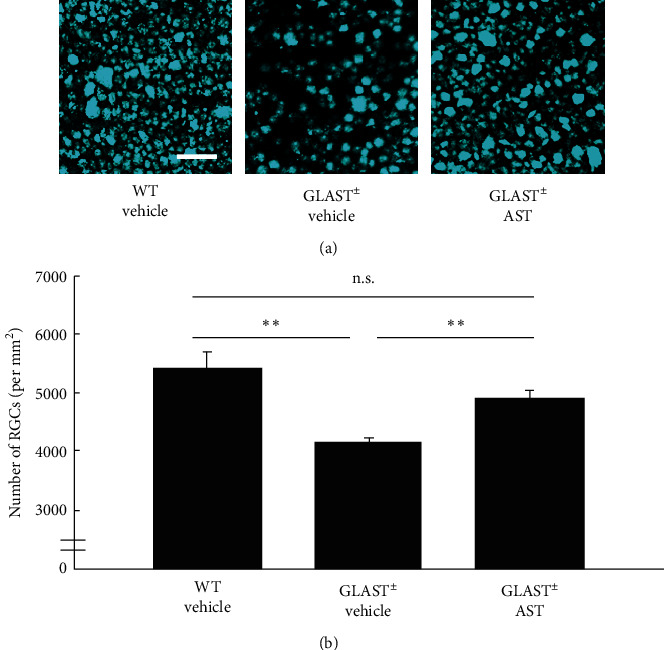
AST-mediated protection of RGCs detected by retrograde labeling. (a) Retrograde-labeled RGCs. Scale bars: 50 *μ*m. (b) Quantification of retrograde-labeled RGCs. Note that the RGC number was significantly lower in Glast ±mice than in control mice, while loss of RGC was suppressed by 60 mg/kg/day AST administration. ^*∗∗*^*P* < 0.01. n.s.: not significant.

To confirm the protection of RGCs by 60 mg/kg/day AST administration, we further examined RGC density using retrograde labeling. Similar to the results of the GCL count, the RGC density in Glast^±^ mice retinas (4159 ± 65 cell number/mm^2^, *n* = 6) was significantly reduced compared with that in WT mice (5423 ± 271 cell number/mm^2^, *n* = 6. *P* < 0.01). This decreased RGC density was similarly suppressed by 60 mg/kg/day AST administration (4923 ± 120 cell number/mm^2^, *n* = 6. *P* < 0.01; Figure 2). Collectively, these data confirm the protective effect of AST against RGC loss in Glast^±^ mice.

### 3.3. IkB Phosphorylation and AST Administration

The transcription factor nuclear factor-*κ*B (NF-*κ*B) is a critical regulator of immune and inflammatory responses [17]. In the normal state, NF-*κ*B binds to the cytoplasmic I*κ*B protein and is inactivated. When I*κ*B is phosphorylated and decomposed, NF-*κ*B is activated and translocates into the nucleus, inducing expression of various target genes [17]. It has been reported that AST suppresses NF-*κ*B activation in the endotoxin-induced uveitis model [18]. We, therefore, calculated the pI*κ*B/I*κ*B ratio to assess NF-*κ*B activity.

**Figure 3:**
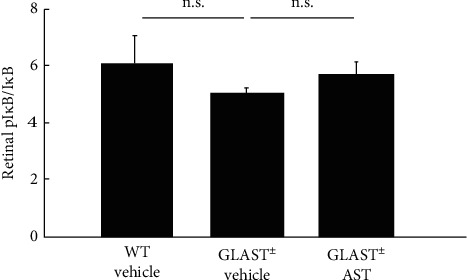
Raito of pI*κ*B/I*κ*B in the AST-administrated retina. Note that the pI*κ*B/I*κ*B ratio was not elevated in Glast^±^mice compared with WT mice and did not change by AST administration. n.s.: not significant.

To measure the activity of NF-*κ*B in Glast^±^ mice, the pI*κ*B/I*κ*B ratio in the retina was calculated. A higher pI*κ*B/I*κ*B ratio indicates an increase in NF-*κ*B pathway activation. Contrary to our expectation, the pI*κ*B/I*κ*B ratio in the retina of Glast^±^ mice did not significantly differ from that of WT mice and was not altered by AST administration (*n* = 7-8 in each group; Figure 3).

### 3.4. Suppression of Oxidative Stress by AST Administration

**Figure 4:**
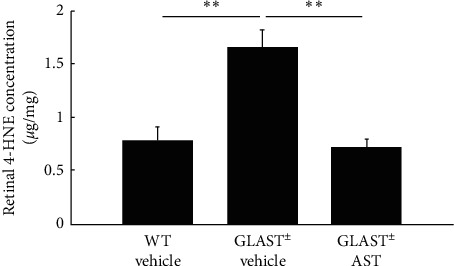
Retinal concentration of 4-HNE after AST administration. Note that the concentration of 4-HNE was elevated in Glast^±^mice compared with WT mice, but was significantly suppressed following AST administration. ^*∗∗*^*P* < 0.01.

To explore the underlying mechanism through which AST inhibited RGC loss, we measured an oxidative stress marker, 4-HNE, in the retina. 4-HNE levels were found to be significantly higher in Glast^±^ mice administered with vehicle (4.5 ± 0.6 *μ*g/mg, *n* = 8) than in WT mice (2.7 ± 0.4 *μ*g/mg, *n* = 8. *P* < 0.01; Figure 4). Additionally, this increase in the 4-HNE level in Glast^±^ mice was significantly suppressed by AST administration (3.0 ± 0.2 *μ*g/mg, *n* = 8. *P* < 0.01; Figure 4).

## 4. Discussion

Herein, we demonstrate that AST prevents glaucomatous retinal degeneration in Glast^±^ mice. Unlike most antioxidants that act in the inner (e.g., vitamin E and *β*-carotene) or the outer (e.g., vitamin C) side of the membrane, AST crosses the bilayer membrane, protecting against oxidative stress by scavenging reactive oxygen species (ROS) in both the inner and outer layers of the cellular membrane [19, 20]. Due to its unique molecular structure, AST exhibits some important biologic properties, mostly represented by the strong antioxidant, anti-inflammatory, and antiapoptotic activities. AST had both antiapoptotic and proapoptotic effects depending on the pathological condition. Indeed, AST has been shown to induce cancer cell apoptosis through a mitochondrial-dependent pathway [21]. On the other hand, AST significantly reduced RGC apoptosis that is responsible for the progression of retinal damage in glaucoma and in other optic neuropathies, as well as RPE cell death that causes AMD development [22, 23].

Furthermore, some studies have indicated that AST prevents RGC death *in vivo* and *in vitro* [24–26]. Otsuka and co-authors suggested that AST inhibited ischemia-induced retinal cell death via its antioxidant effect [24]. Suppressive effects of AST on glaucomatous retinal injury were also assessed by Cort and co-authors [25], who conducted an experimental study on mouse models in which elevated IOP was induced by unilaterally cauterizing episcleral vessels. Lin and co-authors suggested that AST achieved neuroprotective effects in ischemic optic neuropathy model mice by downregulating both oxidative and inflammatory cascades, as AST administration also reduced the expression of TNF*α* and IL1*β* in retinas [26].

This inflammatory cascade is associated with increased oxidative stress. Indeed, cytokines and chemokines induce intracellular ROS generation. These oxidative products, in turn, enhance the inflammatory cascade by NF-*κ*B activation and together alter cellular and molecular targets, destroying normal tissue homeostasis [27, 28]. NF-*κ*B is usually localized in the cytoplasm in an inactivated form and bound to nonphosphorylated I*κ*B*α*. During activation, I*κ*B is phosphorylated and, thus, disassociates from NF-*κ*B. In our study, AST did not decrease the pI*κ*B/I*κ*B ratio in retinas because NF-*κ*B activation was not shown in RGCs of Glast^±^ mice with chronic progressive retinal degeneration, unlike other acute retinal injury models.

Glaucoma is a multifactorial disease. Elevation of IOP is a major risk factor, and the involvement of aging [29], oxidative stress [14, 30, 31], glutamine toxicity [32], and vascular factors [33] has previously been reported. In particular, oxidative stress possibly plays a vital role in the pathogenesis of glaucoma and increases in the antioxidant enzymes superoxide dismutase and glutathione peroxidase have been reported in the aqueous humor of glaucoma patients [31, 34, 35]. Oxidative stress is an important risk factor for human glaucoma [31], and suppression of oxidative stress in RGCs is a potential treatment strategy [36]. In this study, we used Glast^±^ mice as a model of NTG in which Glast, a glutamate transporter that controls extracellular glutamate concentrations, was knocked out. In the retinas of Glast^±^ mice, glutathione concentrations decrease and lipid hydroperoxide concentrations increase, suggesting that NTG-like neurodegeneration may be partially caused by oxidative stress. In addition, IOP in Glast^±^ mice has been reported to be normal [9–11]. In Glast^±^ mice, both increased oxidative stress and glutamate toxicity may contribute to RGC apoptosis [9–11, 37].

In conclusion, we report that the widely prescribed drug AST exerts neuroprotective effects on retinal degeneration in a mouse model of NTG. Our findings indicate that AST may hold therapeutic potential as a novel candidate for the treatment of glaucoma.

## Data Availability

The data supporting the results of the current article are available from the corresponding author upon request.
